# Mental State of the Art: Creative Reflections from Psychiatry Placement

**DOI:** 10.1192/bjo.2025.10265

**Published:** 2025-06-20

**Authors:** Sian Davies, Roshni Bahri, Katie Dichard-Head, Azjad Elmubarak, Ahmad Kamal Mohamed

**Affiliations:** Birmingham and Solihull Mental Health Foundation Trust, Birmingham, United Kingdom

## Abstract

**Aims:** Medical training, particularly in psychiatry, often confronts students with emotional challenges that extend beyond clinical skills. Recognizing that medicine is as much an art as it is a science, we invited fourth year medical students at the University of Birmingham to enter a creativity competition whilst on their psychiatry placements.

This project aimed to provide a reflective outlet for the students, encouraging them to explore and express their emotional responses to the realities of psychiatric practice. By drawing parallels between their own experiences and those of their patients, the students were able to utilise art as a source of personal insight on their clinical practice.

**Methods:** During their 5 week psychiatry rotation, students were invited to create and submit an artistic piece inspired by their clinical encounters. Creative submissions – ranging from poetry and paintings to drawings and even baking – were accompanied by a brief explanation of the inspiration behind the work. On the final day of placement, students had the opportunity to present or perform their piece in front of their peers, fostering an environment of shared reflection and support. One entry in each rotation would be selected as a prize winner, however the main focus of the project was to encourage students to utilise art as a medium of self-reflection and therefore understanding what impact the process had on them was key.

**Results:** 23 students participated in the initiative, producing a diverse collection of artworks that authentically portrayed their personal and professional experiences with mental health. Extracts from the explanations that accompanied the entries highlight that the students benefited from using art as a medium for processing and communicating complicated feelings about their psychiatric placement. The students reflected that art can be used as a therapeutic tool for both patients and clinicians. Many used their creative expression to consider the importance of seeing beyond the label of a diagnosis and to focus on the actual lived experience of the patient in front of us.

**Conclusion:** The creativity competition was met with enthusiasm, underscoring the value of artistic expression in medical education. Moreover, it suggested that integrating creative projects into clinical rotations can enrich students’ learning experiences and bolster empathy. It also encouraged students to continue to use creativity as an outlet to improve their own wellbeing in busy clinical periods. Future plans include publishing the entries in an anthology to share the moving artwork with a wider audience.

